# Latent profiles of problem-solving skills and their association with depressive symptoms in parents of children with cancer: A cross-sectional study

**DOI:** 10.1016/j.apjon.2024.100633

**Published:** 2024-11-30

**Authors:** Tianji Zhou, Yuanhui Luo, Wenjin Xiong, Zhenyu Meng, Nancy Xiaonan Yu, Jingping Zhang

**Affiliations:** a Xiangya School of Nursing, Central South University, Changsha, Hunan, China; b Department of Social and Behavioural Sciences, City University of Hong Kong, Hong Kong, China

**Keywords:** Childhood cancer, Parents, Problem-solving skills, Latent profile analysis, Depressive symptoms

## Abstract

**Objective:**

Depressive symptoms are prevalent among parents of children with cancer, significantly impacting their well-being. Problem-solving skills, strongly linked to depressive symptoms, offer a promising avenue for intervention. This study aimed to identify latent profiles of parental problem-solving skills and evaluate differences in depressive symptoms across these profiles.

**Methods:**

A cross-sectional survey was conducted with 318 parents of children with cancer in mainland China. Self-reported data on demographics, problem-solving skills, and depressive symptoms were collected. Latent profile analysis was used to classify parental problem-solving skills into distinct profiles, and multiple logistic regression identified predictors of profile membership.

**Results:**

Three profiles of problem-solving skills were identified: (1) problem-oriented and constructive (*n* ​= ​94, 29.6%), (2) impulsivity-oriented and irrational (*n* ​= ​76, 23.9%), and (3) emotion-oriented and avoidant (*n* ​= ​148, 46.5%). Parents with higher education, greater income, and urban residency were more likely to belong to the problem-oriented group. Fathers predominated in the impulsivity-oriented group, while mothers were more represented in the emotion-oriented group. Significant differences in depressive symptoms were observed across profiles, with the problem-oriented group reporting the lowest levels.

**Conclusions:**

This study highlights the heterogeneity of problem-solving skills among parents of children with cancer and underscores the need for tailored interventions. Addressing specific characteristics of each profile can improve parental well-being and provide targeted support for this vulnerable population.

**Trial registration:**

ChiCTR2300071828.

## Introduction

Despite advances in medical treatment that have enhanced the 5-year survival rates for childhood cancer,^1^ cancer continues to be the primary disease-related cause of mortality in children,^2^ with around 400,000 new cases identified each year worldwide.^3^ This perilous illness and its long-lasting treatments pose harrowing challenges for both children and their families, particularly for parents who are generally the primary caregivers.^4^ Parents caring for a child with cancer face immense stress due to the high caregiving demands together with the uncertainty of cancer prognosis and the burden of financial difficulties,^5^^,^^6^ which may exacerbate parental psychological distress and potentially lead to mental health issues.^6^ Depressive symptoms appear to be the most frequently reported negative affectivity among parents of children with cancer,^7^ with up to 74.3% of the parents experiencing depressive symptoms.^8^^,^^9^ These negative short-term and long-term impacts of childhood cancer on parents have garnered growing attention, with numerous studies amply demonstrating the significance of comprehensive treatment and nursing support for parents.^10^^,^^11^ Given that parental depressive symptoms greatly impact the quality of life for both parents and their children,^12^^,^^13^ identifying protective factors for intervention that can alleviate depressive symptoms in these parents is essential for enhancing the well-being of both parents and children.

Problem-solving is a cognitive–behavioral process where individuals perceive and implement adaptive solutions to manage everyday challenges and significant stressors.^14^ Evidence shows that problem-solving skills (PSS) are among the most common coping strategies for emotion regulation, whereas deficiencies in this capacity are linked to the onset and continuation of depressive symptoms.^15^ Therefore, improving PSS could enable individuals to identify problem-focused strategies for managing stressful events and thus reduce negative affectivity by effectively solving problems.^14^ In the field of childhood cancer, parental PSS has been consistently associated with the psychosocial well-being of the parents, their children, and the entire family unit when dealing with childhood cancer.^16^^,^^17^ Previous research has also demonstrated a negative correlation between PSS and maladaptive outcomes (anxiety, depression, and posttraumatic stress symptoms) in parents of children with cancer.^18^^,^^19^ Moreover, facilitating problem-solving is highly recommended in current clinical practice guidelines and indicated treatment programs to help manage depressive symptoms,^15^^,^^16^^,^^20^ as applying such skills to effectively address children's disease-related problems may reduce parental depressive symptoms and enhance functioning. Overall, the development of PSS may have synergistic beneficial effects on alleviating parental depressive symptoms.

Currently, the identification and assessment of PSS levels in parents of children with cancer primarily depend on self-report scales by calculating a total scale score.^18^^,^^21^ However, this variable-centered approach, assuming homogeneity in parental PSS,^22^ fails to distinguish subgroups with distinct PSS traits or provide specific intervention recommendations. Additionally, previous studies have exhibited inconsistency in establishing a definitive threshold for PSS. Evidence indicates that PSS encompasses diverse attributes such as rationality, impulsivity/carelessness, and avoidance, and individuals may exhibit distinct characteristics depending on the specific problem they encounter.^14^^,^^23^ Research on parents of children with cancer has also documented varying adjustment patterns of resilience, distress, and posttraumatic growth,^19^^,^^24^ theoretically indicating distinct PSS characteristics among these parents as their problem-solving coping is strongly associated with stress and growth responses. Moreover, tailored interventions and supportive care based on individual characteristics have been strongly emphasized, enhancing treatment effectiveness and contributing to optimal clinical outcomes.^25^^,^^26^ Conversely, a one-size-fits-all coping or emotional intervention may overlook the unique coping mechanisms and psychological profiles of different parents, leading to ineffective or even counterproductive interventions. For instance, parents exhibiting high levels of avoidance might require interventions that gradually expose them to stressors in a supportive environment, whereas structured decision-making strategies may potentially exacerbate their distress. The lack of specificity in interventions may also result in resource misallocation, where support services are not effectively utilized to address the needs of distinct subgroups.^27^ Therefore, it is of utmost importance to evaluate the PSS characteristics of parents of children with cancer to develop individualized interventions that can reduce depressive symptoms and enhance psychological well-being.

In the context of pediatric cancer, nurses are essential in delivering high-quality care for both children and parents.^10^ They often serve as the initial point of contact for families and are the health care professionals who interact most frequently with parents of children undergoing cancer treatment, acting as invaluable resources for those who may feel mentally and physically overwhelmed.^28^ Multiple studies have shown that nurses play a pivotal role in assisting parents as they face and resolve various problems related to the complexities of cancer treatment within the hospital setting, being particularly adept at identifying and addressing the unmet needs of these caregivers during the problem-solving process.^10^^,^^29^ Therefore, by understanding the various PSS traits among parents of children with cancer, nurses can customize their support and interventions to align with the specific coping mechanisms and emotional needs of parents. This personalized approach not only enhances the well-being of the parents but also improves the overall quality of care provided to the children affected by cancer.

Latent profile analysis (LPA) is a person-centered analytic method that classifies individuals with similar attributes into the same group based on patterns of scores across multivariate classification data.^30^ LPA examines participants from the perspective of individualization to intuitively show the heterogeneity across different subgroups and homogeneity within each subgroup based on a probabilistic classification model.^31^ Its application in psychological investigation and behavioral analysis has garnered growing attention,^32^^,^^33^ providing new insights into symptom classification and making it possible to “suit the remedy to the case”. In recent years, several studies have utilized LPA to identify patterns of symptom clusters among children with cancer and psychological distress experienced by their parents, which contribute to a better understanding of subgroup-specific effects on the well-being of both children and their parents and facilitated the identification of demographic variations between subgroups, thereby enabling the development of targeted interventions.^24^^,^^34^, ^35^, ^36^ These findings may provide a comprehensive approach and unique insights into understanding the complex PSS profiles and their characteristics among the parents in the current study. However, to the best of our knowledge, no study has yet examined the latent profiles of PSS in parents of children with cancer, making it difficult to comprehend the characteristics of parental PSS and hindering specific reference for future intervention or prevention efforts. Therefore, this study aimed to (1) explore the latent profiles of PSS in parents of children with cancer using LPA; (2) examine the demographic characteristics associated with these profiles; and (3) compare the parental depressive symptoms of each latent profile of PSS. The results of this study will provide valuable insights and recommendations for developing targeted interventions aimed at improving the psychological well-being of parents of children with cancer. This, in turn, may ultimately enhance the quality of nursing provided in oncology settings, reinforcing the importance of integrating psychological support for parents and caregivers into the comprehensive nursing of childhood cancer.

## Methods

### Design and participants

A cross-sectional study was conducted between May and November 2023, following the STROBE checklist for reporting findings. A convenience sampling method was employed to recruit parents of children with cancer from three tertiary hospitals in Hunan Province, mainland China. Eligible participants included those who: (1) were 18 years or older; (2) have a child aged 0–14 years who had received a cancer diagnosis; (3) were either the mother or father of the child; and (4) could speak and read Chinese. Parents with cognitive impairments or severe mental disorders were excluded from the study. To ensure adequate data aggregation and identification of small profiles, a minimum sample size of 300 cases was recommended for LPA.^37^ In this study, a total of 318 participants were included, providing sufficient power for LPA.

### Measures

#### Demographic characteristics

A self-compiled demographic information sheet was utilized to gather data on participants' demographic characteristics and their children's clinical information. The questionnaire included items related to the parent's gender, age, marital status, educational level, monthly income, place of residence, and number of children. Additionally, participants were asked to provide information on their children's gender, age, type of cancer, risk stratification of cancer, type of treatment, and time since diagnosis. The risk stratification of childhood cancer was identified from children's electronic medical records, which were categorized as low risk, intermediate risk, high risk, and unknown based on the treatment exposures and diagnoses.^38^

#### Social problem-solving inventory-revised: short form (SPSI-R:S)

The PSS of the participants was assessed using the SPSI-R:S, a self-rating questionnaire developed by D'Zurilla and Nezu.^14^^,^^23^ The Chinese version was translated and revised by Siu and Shek, and it has demonstrated satisfactory validity and reliability.^39^ The Chinese version of SPSI-R:S comprises 25 items that are divided into five subscales: positive problem orientation (PPO), negative problem orientation (NPO), rational problem-solving (RPS), impulsivity/carelessness style (ICS), and avoidance style (AS). Participants rated each item on a five-point Likert scale (0 ​= ​“Not at all true of me” to 4 ​= ​“Extremely true of me), with higher scores on each subscale indicating a greater intensity of that dimension. Overall, higher scores on the PPO and RPS subscales, and lower scores on the NPO, ICS, and AS subscales imply better PSS. In the current sample, the Cronbach's α for the total scale and five subscales were 0.903, 0.696, 0.881, 0.879, 0.854, and 0.835, respectively.

#### Patient health Questionnaire-9 (PHQ-9)

The PHQ-9 was used to evaluate the depressive symptoms experienced by the participants during the last two weeks.^40^ The Chinese version of this scale has been well-validated in multiple studies, presenting a high level of internal consistency and reliability.^41^^,^^42^ The PHQ-9, widely used for screening depressive symptoms globally, comprises nine items, and each item is rated on a four-point scale from 0 (not at all) to 3 (nearly every day). The total score on the PHQ-9 is calculated by summing the scores of individual items, ranging from 0 to 27, with higher scores indicating more severe depressive symptoms. A cut-off of 10 points has been suggested, where a total score of 0–4 indicates minimal or none; 5–9, mild; 10–14, moderate; 15–19, moderately severe; and 20–27, severe depression.^40^ The Cronbach's α for this scale in this study was 0.700.

### Data collection

Systematically trained researchers visited the department of pediatric hematology and oncology in the three hospitals to invite eligible parents to participate in the study. The researchers provided detailed information about the purpose and voluntary nature of the study to the eligible parents. Those who agreed to participate signed an informed consent form and were then given self-report questionnaires for data collection, which typically took around 10–15 minutes to fill out. All data collection was conducted face-to-face, and participants were provided with sufficient time and privacy to complete their filling process independently. Researchers were available to answer questions carefully and assist participants according to their requirements when necessary. After participants completed the survey, the researchers carefully checked all questionnaires to ensure the completeness of the information provided.

### Data analysis

The self-report approach was employed for data collection in this study, which potentially introduced a risk of common method bias. To address this concern, Harman's single-factor method was used.^43^ In this test, all items from the SPSI-R:S and PHQ-9 scales underwent exploratory factor analysis. A single factor extraction yielding values greater than 40% of the total variance indicates a possible presence of common method bias.

LPA was conducted using Mplus version 8.3. The observable indicators for LPA were the five dimensions of the SPSI-R:S, and a series of models were estimated by gradually increasing the profile number from the initial (one profile) to the final model (five profiles) until the optimal fit metrics were achieved. Multiple fit metrics were considered to facilitate the model choice, including the commonly used information indicators: Akaike information criterion (AIC), Bayesian information criterion (BIC), and sample-size-adjusted BIC (aBIC). Lower values on these metrics indicate a better fit for the model.^44^ The entropy value represents the classification accuracy, with values above 0.80 generally considered acceptable.^45^ Additionally, the Lo-Mendell-Rubin Likelihood Ratio Test (LMRT) and Bootstrap Likelihood Ratio Test (BLRT) were used to compare models with continually increasing profile numbers. A significant result on these tests (*P* ​< ​0.05) indicates that the k profiles model fits better than the k-1 profiles.^46^ The interpretability and parsimony of the profiles were also taken into account when selecting the optimal model.

Other data analyses were conducted using SPSS version 26.0 (IBM Corporation, Armonk, New York, USA). The normality of continuous variables was assessed using the Kolmogorov–Smirnov test, which indicated that the key variables included in this study were normally distributed. Descriptive statistics, such as mean and standard deviation (SD) for continuous variables and frequency and percentage for categorical variables, were calculated. On top of determining the optimal model, the demographic characteristics and inter-group comparisons were analyzed using the χ^2^ test or Fisher's exact probability method. The statistically significant indicators identified from univariate analysis were included in multinomial logistic regression to explore the predictors of the latent profiles of PSS. Finally, one-way ANOVA and the Student–Newman–Keuls (SNK) post hoc tests were performed to compare the depressive symptoms experienced by parents in each latent profile. The significance level was set at *P* ​< ​0.05 (two-tailed).

### Ethical considerations

The study was approved by the Institutional Review Board of Xiangya School of Nursing, Central South University (IRB No. E2023113). Prior to participating in the study, all parents signed an informed consent form and were provided with comprehensive information about the purpose and procedures of the study. Additionally, parents were assured that they had the right to withdraw from the survey at any time without any impact on their child's treatment. All data collected were anonymized with numbers and stored in a confidential computer, accessible only to the researchers.

## Results

### Common method bias test

The results showed that three factors with eigenvalues greater than 1 were extracted, and the first factor accounted for 29.796% of the total variance. As this percentage falls below the 40% threshold, it suggests that there is no significant common method bias present in this study.

### Samples characteristics

A total of 350 participants were invited to take part in this study, with 28 parents opting out due to busy schedules or disinterest. Additionally, four questionnaires that were mostly incomplete were excluded from the analysis, leading to a response rate of 90.86%. Ultimately, 318 parents of children with cancer (219 mothers and 99 fathers) were included in the study, with an average age of 35.75 years (SD ​= ​5.28 years). The majority were married (93.4%) and resided in rural areas (74.5%). More than half of the parents had completed a high school education (57.9%) and had two children (56.0%). Approximately three-quarters (78.0%) of the participants’ children had been diagnosed with hematological malignancies, while the remaining 22.0% had solid tumors. Of the 318 children of the included parents, 171 (53.8%) were male and 147 (46.2%) were female. The mean age of the children was 6.91 years (SD ​= ​3.62 years), with nearly half of the children (47.2%) having received their diagnoses within the last six months. Further details regarding the demographic characteristics are presented in Table 1.

**Table 1:** Demographic characteristics and univariate analysis of latent profiles.

Variables	Overall (*n* ​= ​318) *n* (%)	Profile1 (*n* ​= ​94) *n* (%)	Profile 2 (*n* ​= ​76) *n* (%)	Profile 3 (*n* ​= ​148) *n* (%)	*P*
**Parents**					
Sex					
Male	99 (31.1)	22 (23.4)	71 (93.4)	6 (4.1)	< 0.001∗∗∗
Female	219 (68.9)	72 (76.6)	5 (6.6)	142 (95.9)	
Age (years)					
< ​30	33 (10.4)	6 (6.4)	5 (6.6)	22 (14.9)	< 0.001∗∗∗
30–40	226 (71.1)	83 (88.3)	42 (55.3)	101 (68.2)	
> ​40	59 (18.6)	5 (5.3)	29 (38.2)	25 (16.9)	
Marital status					
Married	297 (93.4)	93 (98.9)	69 (90.8)	135 (91.2)	0.036∗
Unmarried/widowed/divorced	21 (6.6)	1 (1.1)	7 (9.2)	13 (8.8)	
Education level					
Primary school	41 (12.9)	3 (3.2)	13 (17.1)	25 (16.9)	< 0.001∗∗∗
High school	184 (57.9)	33 (35.1)	51 (67.1)	100 (67.6)	
College	93 (29.2)	58 (61.7)	12 (15.8)	23 (15.5)	
Monthly income (¥, CNY^a^)					
< ​3000	122 (38.4)	11 (11.7)	34 (44.7)	77 (52.0)	< 0.001∗∗∗
3000–5000	105 (33.0)	18 (19.1)	26 (34.2)	61 (41.2)	
> ​5000	91 (28.6)	65 (69.1)	16 (21.1)	10 (6.8)	
Place of residence					
Rural	237 (74.5)	39 (41.5)	70 (92.1)	128 (86.5)	< 0.001∗∗∗
Urban	81 (25.5)	55 (58.5)	6 (7.9)	20 (13.5)	
Number of children					
1	88 (27.7)	25 (26.6)	24 (31.6)	39 (26.4)	0.046∗
2	178 (56.0)	62 (66.0)	38 (50.0)	78 (52.7)	
≥ ​3	52 (16.4)	7 (7.4)	14 (18.4)	31 (20.9)	
**Children with cancer**					
Type of cancer					
Hematological malignancy	248 (78.0)	70 (74.5)	60 (78.9)	118 (79.7)	0.620
Solid tumor	70 (22.0)	24 (25.5)	16 (21.1)	30 (30.3)	
Risk stratification of cancer					
Low	68 (21.4)	22 (23.4)	20 (26.3)	26 (17.6)	0.423
Intermediate	105 (33.0)	36 (38.3)	20 (26.3)	49 (33.1)	
High	88 (27.7)	20 (21.3)	23 (30.3)	45 (30.4)	
Unknown	57 (17.9)	16 (17.0)	13 (17.1)	28 (18.9)	
Type of treatment					
Single therapy	224 (70.4)	61 (64.9)	53 (69.7)	110 (74.3)	0.307
Multiple therapies	82 (25.8)	29 (30.9)	18 (23.7)	35 (23.6)	
Unknown	12 (3.8)	4 (4.3)	5 (6.6)	3 (2.0)	
Time since diagnosis (months)					
< ​6	150 (47.2)	40 (42.6)	44 (57.9)	66 (44.6)	0.251
6–12	110 (34.6)	37 (39.4)	19 (25.0)	54 (36.5)	
> ​12	58 (18.2)	17 (18.1)	13 (17.1)	28 (18.9)	

∗*P* ​< ​0.05 (2-tailed); ∗∗*P* ​< ​0.01 (2-tailed); ∗∗∗*P* ​< ​0.001 (2-tailed).

Profile 1, Problem-oriented and constructive problem-solving; Profile 2, Impulsivity-oriented and irrational problem-solving; Profile 3, Emotion-oriented and avoidant problem-solving.

a CNY = China Yuan, US$ 1.00 ​= ​¥ 7.02.

### Results of latent profile analysis

In this study, the potential profiles of the five dimensions of PSS in parents of children with cancer were analyzed. Five models (profiles 1–5) were successively fitted, and Table 2 shows the fit metrics for the LPA model, with the best values for each respective statistic highlighted in bold. The results indicated that as the number of profiles increased, the values of AIC, BIC, and aBIC gradually decreased. The *P*-values for LMRT and BLRT were consistently less than 0.05 across all fitted models, and the three-profile model exhibited the highest Entropy value (0.938). Additionally, there were large decreases in model fit metrics (AIC, BIC, and aBIC) from the one-profile model to the three-profile model, while the decreases in these metrics were small from the three-profile model onward. It is very common that fit indices continue to decline with additional profiles added. Analyzing the range of decreases is useful for identifying an elbow point for the “diminishing returns” (e.g., small decreases in BIC) of model fit improvement.^37^ Therefore, the three-profile LPA model was selected as the final fitted model, which demonstrates high classification quality with average posterior probabilities ranging from 0.963 to 0.984.

**Table 2:** Latent profile analysis model fit metrics.

Profile	k	AIC	BIC	aBIC	Entropy	LMR (*P*)	BLRT (*P*)	Profile proportions	AvePP
1	10	8702.098	8739.719	8708.001	–	–	–	–	–
2	16	8355.601	8415.794	8365.046	0.790	**< 0.001**	**< 0.001**	0.535/0.465	0.935–0.945
3	22	7917.934	8000.699	7930.920	**0.938**	**< 0.001**	**< 0.001**	0.465/0.239/0.296	**0.963–0.984**
4	28	7763.399	7868.737	7779.927	0.913	**0.0404**	**< 0.001**	0.160/0.270/0.233/0.337	0.921–0.989
5	34	**7618.011**	**7745.920**	**7638.080**	0.913	**0.0090**	**< 0.001**	0.207/0.296/0.236/0.104/0.157	0.914–0.984

k, Number of free parameters; AIC, Akaike information criterion; BIC, Bayesian information criteria; aBIC, sample-size-adjusted Bayesian information criteria; LMRT, Lo-Mendell-Rubin Likelihood Ratio Test; BLRT, Bootstrap Likelihood Ratio Test; Avepp, Average posterior probabilities.

**Bolded** values indicate the “best” fit for each respective statistic.

### Naming of latent profile

**Fig. 1:**
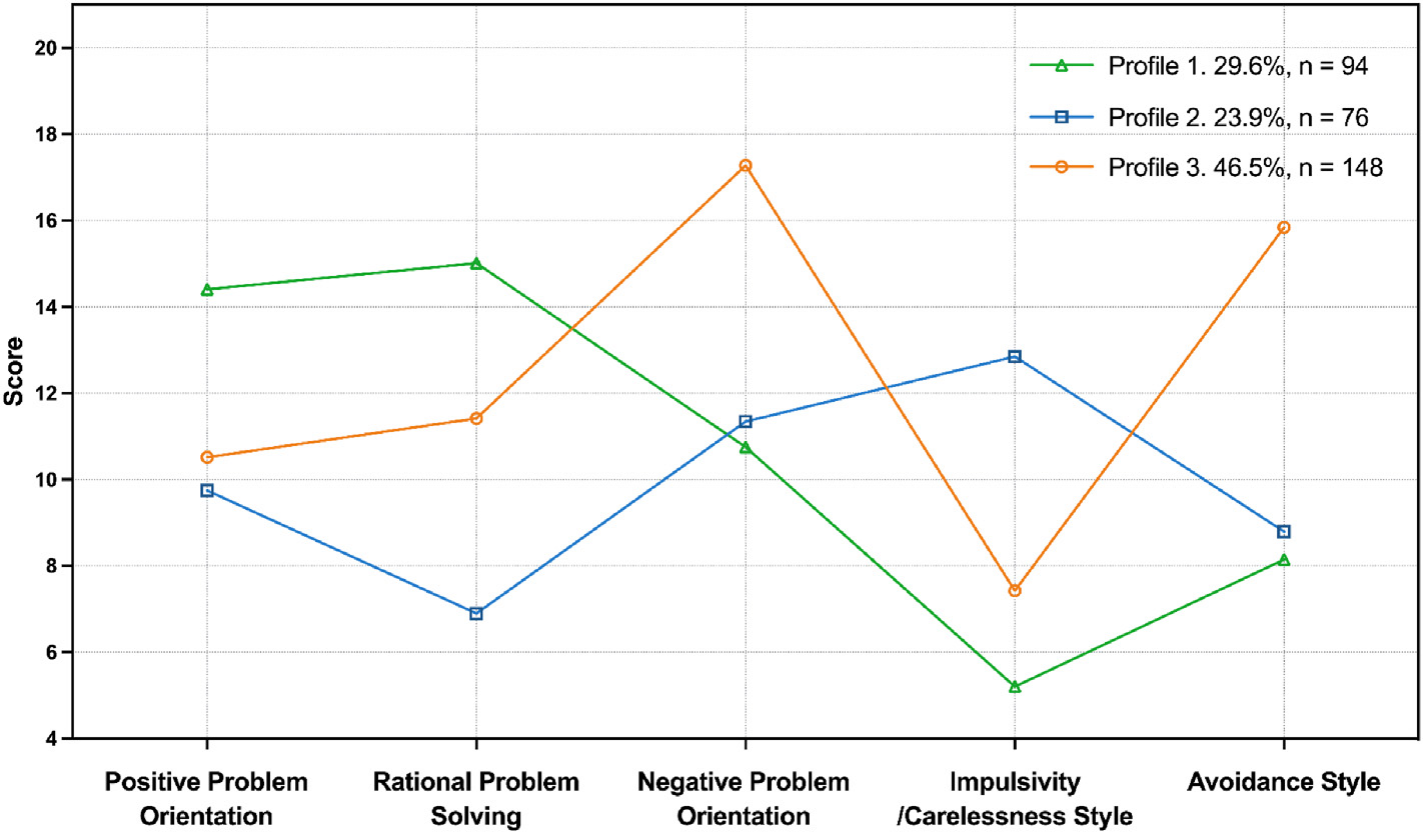
*Latent profiles of problem-solving skills in parents of children with cancer* Profile 1, Problem-oriented and constructive problem-solving; Profile 2, Impulsivity-oriented and irrational problem-solving; Profile 3, Emotion-oriented and avoidant problem-solving.

The scores of the three profiles on the five dimensions of PSS are plotted in Fig. 1. Profile 1 demonstrated significantly higher scores in positive problem orientation and rational problem-solving, and had lower scores in negative problem orientation, impulsivity/carelessness style, and avoidance style compared to Profile 2 and Profile 3, comprising 29.6% (*n* ​= ​94) of the participants, and this profile was named “problem-oriented and constructive problem-solving”. Profile 2 was characterized by the lowest score on rational problem-solving and the highest score on impulsivity/carelessness style among the three profiles, so it was named “impulsivity-oriented and irrational problem-solving”, accounting for 23.9% (*n* ​= ​76) of the parents. Finally, Profile 3, representing the remaining 46.5% (*n* ​= ​148) of the participants, received the highest scores in negative problem orientation and avoidance style compared to the other profiles, leading to the name “emotion-oriented and avoidant problem-solving”.

### Inter-profile characteristic differences

Table 1 presents the differences in demographic characteristics among the different profiles. The results showed that, except for children's clinical information, statistically significant differences were observed in all parents' demographic indicators (all *P*-values ​< ​0.05). Specifically, the proportion of parents aged between 30 and 40, with a college degree, having a monthly income of > 5000 yuan, and living in urban areas was highest in the “problem-oriented and constructive problem-solving” group. It is noteworthy that participants in the “impulsivity-oriented and irrational problem-solving” group were mostly fathers of children with cancer (93.4%), while in the “emotion-oriented and avoidant problem-solving” group, the majority were mothers (95.9%).

### Multinomial logistic regression of latent profiles

A multiple logistic regression analysis was conducted to assess the predictive impact of statistically significant variables in univariate analysis on each profile with Profile 1 as a reference (the results for Profile 2 compared with Profile 3 are presented in Supplementary file 1). Table 3 shows that parents’ gender, age, education level, monthly income, and place of residence were significantly associated with the profile membership. Compared to Profile 1, fathers had a higher likelihood of being in Profile 2 (OR ​= ​56.080, *P* ​< ​0.001) and a lower likelihood of being in Profile 3 (OR ​= ​0.097, *P* ​= ​0.001). Compared with parents aged 40 or above, parents aged between 30 and 40 had a lower probability of belonging to either Profile 2 (OR ​= ​0.139, *P* ​= ​0.022) or Profile 3 (OR ​= ​0.155, *P* ​= ​0.015). Compared to those with a college education, participants with only a primary school education were more likely to be in Profile 3 (OR ​= ​9.930, *P* ​= ​0.011), and those with a high school degree had a higher probability of being in either Profile 2 (OR ​= ​3.899, *P* ​= ​0.045) or Profile 3 (OR ​= ​5.508, *P* ​< ​0.001) than Profile 1. Additionally, compared to a monthly income of > 5000 yuan, parents with a monthly income of < 3000 or between 3000 and 5000 were inclined to belong to Profile 2 (OR ​= ​6.703, *P* ​= ​0.007; OR ​= ​5.690, *P* ​= ​0.012) and Profile 3 (OR ​= ​75.027, *P* ​< ​0.001; OR ​= ​31.473, *P* ​< ​0.001). Compared with urban areas, parents residing in rural areas were 8.4 times more likely to belong to Profile 2 than Profile 1 (OR ​= ​8.370, *P* ​= ​0.002) and 2.6 times more likely to fall under Profile 3 than Profile 1 (OR ​= ​2.600, *P* ​= ​0.041).

**Table 3:** Multinomial logistic regression of latent profiles.

Variables	Profile 2 *vs*. Profile 1			Profile 3 *vs*. Profile 1		
	OR	95% CI	*P*	OR	95% CI	*P*
Sex (ref: female)						
Male	56.080	14.262–220.517	< 0.001∗∗∗	0.097	0.024–0.390	0.001∗∗
Age (ref: > 40)						
< ​30	0.401	0.029–5.584	0.497	0.643	0.094–4.402	0.653
30–40	0.139	0.026–0.753	0.022∗	0.155	0.035–0.692	0.015∗
Marital status (ref: unmarried/widowed/divorced)						
Married	0.412	0.027–6.269	0.523	0.575	0.057–5.842	0.640
Education level (ref: college)						
Primary school	3.553	0.499–25.318	0.206	9.930	1.706–57.813	0.011∗
High school	3.899	1.033–14.714	0.045∗	5.508	2.118–14.322	< 0.001∗∗∗
Monthly income (¥, CNY^a^) (ref: > 5000)						
< ​3000	6.703	1.698–26.464	0.007∗∗	75.027	21.569–260.971	< 0.001∗∗∗
3000–5000	5.690	1.465–22.097	0.012∗	31.473	10.578–93.638	< 0.001∗∗∗
Place of residence (ref: urban)						
Rural	8.370	2.127–32.936	0.002∗∗	2.600	1.040–6.495	0.041∗
Number of children (ref: ≥ 3)						
1	0.850	0.119–6.087	0.872	0.594	0.122–2.877	0.517
2	0.240	0.043–1.348	0.105	0.492	0.128–1.895	0.303

∗*P* ​< ​0.05 (2-tailed); ∗∗*P* ​< ​0.01 (2-tailed); ∗∗∗*P* ​< ​0.001 (2-tailed).

Profile 1, Problem-oriented and constructive problem-solving; Profile 2, Impulsivity-oriented and irrational problem-solving; Profile 3, Emotion-oriented and avoidant problem-solving.

Ref, Reference; SE, Standard error; OR, Odds ratio.

a CNY = China Yuan, US$ 1.00 ​= ​¥ 7.02.

### Depressive symptoms across latent profiles

The mean depressive symptoms scores (SD) for parents in Profile 1–3 were 6.39 (2.04), 10.70 (2.61), and 12.14 (2.94), respectively. According to the PHQ-9 cut-off scores, only parents in Profile one exhibited mild depressive symptoms, while those in Profiles 2 and 3 reported experiencing moderate depressive symptoms. The results of ANOVA indicated significant differences in depressive symptoms across the three latent profiles (*F* ​= ​140.836, *P* ​< ​0.001). The post hoc SNK test indicated that participants in Profile 1 had significantly lower depressive symptom scores than those in Profiles 2 and 3, with Profile 3 displaying the highest levels. These findings are detailed in Table 4.

**Table 4:** Comparison of depressive symptoms among latent profiles.

	Mean ​± ​SD	*P*	SNK test
**Depressive symptoms**	10.10 ​± ​3.60	< 0.001	Profile 3 ​> ​Profile 2 ​> ​Profile 1
Profile1 (*n* ​= ​94)	6.39 ​± ​2.04		
Profile 2 (*n* ​= ​76)	10.70 ​± ​2.61		
Profile 3 (*n* ​= ​148)	12.14 ​± ​2.94		

Profile 1, Problem-oriented and constructive problem-solving; Profile 2, Impulsivity-oriented and irrational problem-solving; Profile 3, Emotion-oriented and avoidant problem-solving.

SD, Standard deviation; SNK, Student-Newman-Keuls.

## Discussion

This study explored the characteristics of PSS in parents of children with cancer, a topic that has been relatively underexplored. To the best of our knowledge, this study is the first to use LPA to examine the latent profiles of PSS and their associations with depressive symptoms in this population. Using a person-centered approach, our findings partially aligned with previous research on the relationships between PSS and psychological adjustment outcomes among parents of children with cancer^16^^,^^18^ and extended the existing literature to consider the heterogeneity in PSS. We identified three distinct PSS profiles that could serve as a reference for future research and tailored intervention efforts.

The “problem-oriented and constructive problem-solving” group (Profile 1) comprised 29.6% of the participants and scored positively on all dimensions among the three profiles. Parents belonging to this profile exhibited sufficient confidence to face challenges with a positive attitude and systematically solve problems, which enabled them to cope effectively with daily issues regarding their children's complex treatment process. Positive problem orientation and rational problem-solving were prominent features of parents in this profile. These findings are consistent with previous reports suggesting that parents with greater levels of hope, optimism, and resilience experience higher perceived control of problem–solving, contributing to better adaptation to their children's cancer diagnoses.^19^^,^^47^^,^^48^ However, this does not imply that parents within this profile do not require attention; they still need guidance and encouragement in certain areas to persistently practice and employ PSS when facing stressful conditions to facilitate further development. Moreover, previous studies have shown that peer support from veteran parents of children with cancer to other parents has synergistic benefits for both the veteran parents and those they assist.^49^^,^^50^ Therefore, directing parents to the “problem-oriented and constructive problem-solving” group and encouraging them to support their peers who struggle with similarly intense and ongoing stressors associated with childhood cancers may achieve a win–win situation. In the univariate analysis, parents in this profile outperformed those in the other two profiles regarding demographic factors such as education level, monthly income, and place of residence, which supports the rationale for this grouping.

The “impulsivity-oriented and irrational problem-solving” group (Profile 2), comprising 23.9% of the parents, scored highest in impulsivity/carelessness style and lowest in rational problem-solving. Although parents belonging to this profile are self-motivated to embark on challenges and problems, they are characterized by impulsiveness, carelessness, hastiness, and inadequate attempts during the problem-solving process. One possible explanation is that the high-stress environment of childhood cancer, coupled with the constant need for quick decision-making and ongoing emotional and physical fatigue, may lead to parents' impulsive actions without thorough consideration of all possible solutions.^51^, ^52^, ^53^ Those who employ an impetuous and irrational problem-solving style typically generate limited solution options without comprehensively analyzing their children's disease-related problems and tend to act on the first solution that comes to their minds.^14^^,^^54^ Given that an impulsiveness/carelessness style may perpetuate a repeated process of ineffective problem-solving and increase negative affectivity,^55^^,^^56^ mastering the steps to systematically solve problems, which involves defining and formulating the problem, generating alternative solutions, making decisions, implementing solutions, and evaluating outcomes,^57^ is highly recommended for parents in this profile.

In contrast to Profile 2, the “emotion-oriented and avoidant problem-solving” group (Profile 3), which consisted of 46.5% of the participants, exhibited peak scores on negative problem orientation and avoidance style. Parents in this profile view problems as major threats and unsolvable challenges, and they are prone to avoiding problems, procrastinating, and relying on others to solve their problems. This finding of the current study aligns with previous research stating that avoidance style and distancing coping are more frequently adopted by parents of children with cancer in response to emotional distress.^58^^,^^59^ On the one hand, the profound emotional distress and a lack of control associated with childhood cancer may compel parents to avoid directly facing the harsh reality of the situation.^18^^,^^47^ On the other hand, emotion-focused problem-solving may serve as a coping mechanism to temporarily regulate the emotional consequence of witnessing their children's suffering and the perceived uncertainty of the future, by which parents can shield themselves from the emotional pain.^60^ Furthermore, evidence has indicated that a negative problem orientation and avoidance style can result in higher psychological distress, increased posttraumatic stress symptoms, and lower quality of life levels.^59^^,^^61^ Therefore, for parents in the “emotion-oriented and avoidant problem-solving” group, interventions should be focused on positive problem orientation and character strengths exploration to facilitate parents' initiative to face and solve problems.

The multinomial logistic regression analysis indicated that parents' socioeconomic status significantly predicts profile membership. Specifically, compared to Profile 1, parents with poorer education levels, lower monthly incomes, and those residing in rural areas were more likely to fall into Profile two or Profile 3, both of which are considered dysfunctional problem-solving strategies. This finding is supported by extensive studies, which point out that parents belonging to a lower sociodemographic group report less use of problem-oriented or task-oriented coping due to a lack of self-efficacy and perceived social support, resulting in their poor adaptation.^9^^,^^24^^,^^59^^,^^62^ Therefore, parents of children with cancer might face a higher risk of ineffective problem-solving in the context of low socioeconomic status, highlighting the need for timely screening and targeted support for these parents. Additionally, parents aged between 30 and 40 were more likely to be in the “problem-oriented and constructive problem-solving” group, as younger parents may lack experience and older parents may lack energy due to the overwhelming time and effort required by their children's treatment process,^6^^,^^63^ which impedes their ability to adopt constructive problem-solving approaches. Hence, experienced parents are encouraged to share their proven and replicable solutions to specific and common problems encountered during childhood cancer. Furthermore, we did not find significant associations between children's characteristics and profile membership. While this result does not align with previous findings that children's age and treatment stage often affect parents' levels of distress and coping strategies in the context of childhood chronic disease,^8^^,^^16^ it underscores the extensive and profound impact of childhood cancer on parental PSS.

It is noteworthy that fathers of children with cancer were more likely to be in the “impulsivity-oriented and irrational problem-solving” group, while mothers were more inclined to belong to the “emotion-oriented and avoidant problem-solving” group. Gender differences in problem-solving strategies might be attributed to biological variations in neural response to psychological stress.^64^ Mothers are more often the primary caregivers at the hospital, witnessing the pain and suffering of their children and experiencing substantial disruptions in family and social interactions.^6^ Extensive studies from various cultural backgrounds also demonstrated that mothers frequently withdraw from conversations regarding their child's disease-related issues or postpone critical decisions in order to seek temporary relief from the harsh realities associated with childhood cancer.^60^^,^^65^ Consequently, they are more likely than fathers to exhibit maladaptive behaviors and adopt emotion-focused coping strategies.^19^^,^^66^ Additionally, social norms may shape how fathers, generally the breadwinners of the family, express and report their stress. They tend to prioritize swift problem resolution, sometimes at the expense of thorough analysis and consideration.^67^ However, prior research conducted in developed countries, such as the United States and Sweden, has indicated that fathers often exhibit a more proactive approach to problem-solving, potentially due to differing societal expectations, out-of-pocket medical costs, and support systems.^47^^,^^65^^,^^68^ This may lead to a lower prevalence of impulsivity-oriented profiles among fathers in those cultural contexts. Therefore, in contrast to the traditional focus of problem-solving interventions on mothers of children with cancer,^17^^,^^57^ the present findings suggest that improving PSS could benefit both mothers and fathers since they exhibited dysfunctional problem-solving to a certain degree. Overall, future PSS interventions targeting both mothers (with a focus on establishing a positive problem orientation) and fathers (with an emphasis on mastering the steps to systematically solve problems) should be refined and developed to effectively enhance their well-being.

This study demonstrated significant differences in depressive symptoms among parents with distinct PSS characteristics. The depressive symptoms score for the “problem-oriented and constructive problem-solving” group fell below the diagnostic threshold and was notably lower than the scores of the other two profiles. These findings are consistent with earlier studies, which found that constructive problem-solving can prevent episodes of negative affectivity, whereas dysfunctional problem-solving robustly predicts poorer adjustment outcomes in parents of children with cancer.^18^^,^^47^ Moreover, we found that the “emotion-oriented and avoidant problem-solving” group experienced higher levels of depressive symptoms than the “impulsivity-oriented and irrational problem-solving” group, potentially due to gender and role differences in terms of reporting psychological distress between these two profiles.^66^^,^^67^ Another potential explanation is that negative problem orientation and avoidant coping are inherently accompanied by negative emotions,^69^ thus exacerbating parental depressive symptoms in Profile 3. Such findings further emphasize the significance of tailored interventions based on the different features of parental PSS for alleviating depressive symptoms and improving functioning. Therefore, improving PSS could be an essential and highly promising means of enhancing the well-being of parents of children with cancer.

### Limitations

The study has several limitations that should be acknowledged. First, the study sample comprised parents recruited from three tertiary hospitals within a single province in mainland China through convenience sampling, which may limit the generalizability of our findings to broader populations. Future research should aim to include participants from different regions and diverse cultural backgrounds to gain a deeper understanding. Second, the cross-sectional design of this study limits our capacity to observe changes in profiles and infer causal relationships between parental PSS profiles and depressive symptoms. Future longitudinal research could employ latent transition analysis, a longitudinal extension of LPA,^70^ to investigate shifts between PSS profiles over time and validate their predictive relationship with depressive symptoms. Additionally, a Cronbach's α of 0.700 for PHQ-9 in the current study is frequently regarded as the minimum acceptable threshold for reliability, which indicates the need for caution when interpreting the results associated with depressive symptom scores. Finally, the multinomial logistic regression analysis included only demographic variables, highlighting the necessity for future research to incorporate psychosocial and physical outcomes for a more comprehensive prediction of profile membership.

### Implications for nursing practice and research

This study has significant implications for both the scientific exploration of PSS and its practical applications in nursing care. First, the results underscore the importance of adopting a person-centered approach when investigating PSS in parents of children with cancer. This approach extends related theories and previous studies by illustrating the distinct patterns of PSS identified in the study, which can guide nursing assessments and interventions. Second, health care professionals, particularly clinical nurses, play a crucial role in assessing the mental health status of parents and identifying specific characteristics of their PSS based on demographic predictors. It is essential for nurses to pay particular attention to parents from low socioeconomic backgrounds and to recognize signs of dysfunctional problem-solving behaviors when caring for their children with cancer, such as those observed in Profile 2 (carelessness) and Profile 3 (avoidance). Nurses can also engage in open conversations with parents to understand their unique challenges, concerns, and coping styles related to problem-solving. Early recognition of these signs enables nurses to offer targeted advice and interventions to support parents effectively. Furthermore, as an effective strategy to reduce parental depressive symptoms and improve their well-being, targeted PSS interventions should be developed based on a comprehensive understanding of the characteristics of each PSS profile. For parents in the “problem-oriented and constructive problem-solving” group, nurses could facilitate peer support groups where parents can share their strategies and experiences with other parents, fostering a supportive community that benefits both the parents and their peers. Considering that there is room for improvement in rational problem-solving for parents in the “impulsivity-oriented and irrational problem-solving” group, nursing intervention emphasizing systematic problem-solving steps should be implemented to reduce their impulsivity and carelessness. Nurses can provide structured guidance and resources to help these parents develop more effective coping strategies, thereby reducing impulsivity and carelessness. It is also recommended that emotion management and character strength training be incorporated into the PSS intervention for parents in the “emotion-oriented and avoidant problem-solving” group to help them establish a positive problem orientation when confronted with challenges. For example, nurses could encourage these parents to record three good things daily or share their meaningful experiences through photography within the hospital^71^ to help them foster an active mindset toward their children's cancer-related problems.

The PSS training, recognized as an evidence-based cancer control program, has proven effective in enhancing parental PSS and reducing depressive symptoms in Western countries.^16^^,^^57^ The training may serve as an emerging and effective intervention for the current study, consisting of two key components: fostering a positive problem orientation and mastering systematic problem-solving steps.^17^ These two components emphasize equipping parents with the skills to face problems positively and solve them carefully, which are helpful in addressing parents' avoidance style in Profile 3 and impulsivity style in Profile two in a targeted manner. Although the effectiveness of PSS training is well established, applying it in the Chinese context is still at an exploratory stage.^72^ Therefore, the present study suggests that refining PSS training by emphasizing cultural appropriateness and tailoring it to the characteristics of each profile is crucial for further enhancing PSS in parents of children with cancer. Clinical nurses and nursing professionals are encouraged to translate this evidence-based intervention into nursing practice and incorporate it as an active component of psychosocial interventions to better support the psychological well-being of parents during their children's cancer treatment. For instance, nurses may facilitate group workshops in which parents can participate in PSS training sessions to overcome their dysfunctional problem-solving styles, with a focus on typical challenges faced by parents while providing care in a hospital setting.

## Conclusions

This study identified three latent profiles of PSS in parents of children with cancer, and there were differences in gender and socioeconomic status among different profiles of parents. Health care professionals, particularly clinical nurses, should pay special attention to parents in the “impulsivity-oriented and irrational problem-solving” and “emotion-oriented and avoidant problem-solving” groups, who exhibited dysfunctional problem-solving strategies and reported relatively higher levels of depressive symptoms. In conclusion, it is essential to develop and implement targeted interventions tailored to the characteristics of various profiles of PSS in parents of children with cancer to enhance parental PSS and reduce depressive symptoms. Furthermore, PSS training is strongly recommended for these parents and should be refined to address the specific needs of each profile in the context of oncology nursing.

## CRediT authorship contribution statement

Tianji Zhou: Writing – Original Draft, Visualization, Formal analysis, Funding acquisition, Conceptualization. Yuanhui Luo: Writing – Review & Editing, Methodology, Funding acquisition, Conceptualization. Wenjin Xiong: Writing – Review & Editing, Investigation. Zhenyu Meng: Writing – Review & Editing, Investigation. Nancy Xiaonan Yu: Writing – Review & Editing, Conceptualization. Jingping Zhang: Writing – Review & Editing, Supervision, Project administration, Data Curation. All authors had full access to all the data in the study, and the corresponding author had final responsibility for the decision to submit for publication. The corresponding author attests that all listed authors meet authorship criteria and that no others meeting the criteria have been omitted.

## Ethics statement

The study was approved by the Institutional Review Board of Xiangya School of Nursing, Central South University (IRB No. E2023113). All parents provided written informed consent.

## Funding

This work was supported by the 10.13039/100001547China Medical Board (Grant No. CMB–OC–22-462), the Postgraduate Independent Exploration and Innovation Project of 10.13039/501100002822Central South University (Grant No. 2024ZZTS0542), and 10.13039/501100002767Hunan Provincial Science and Technology Department (Grant No. 2023JJ40795). The funders had no role in considering the study design or in the collection, analysis, interpretation of data, writing of the report, or decision to submit the article for publication.

## Data availability **statement**

The data that support the findings of this study are available from the corresponding author upon reasonable request.

## Declaration of generative AI and AI-assisted technologies in the writing process

No AI tools/services were used during the preparation of this work.

## Declaration of competing interest

The authors declare no conflict of interest.
