# Supplementary material for: Latent profiles of problem-solving skills and their association with depressive symptoms in parents of children with cancer: A cross-sectional study
Source: Asia Pac J Oncol Nurs. 2024 Nov 30;12:100633. doi: 10.1016/j.apjon.2024.100633 (PMC11699806; doi:10.1016/j.apjon.2024.100633)
Supplement: Multimedia component 1 [file mmc1.docx]

**Multinomial logistic regression of latent profiles (Profile 3 VS. Profile 2)**

| **Variables** | **Profile 3 Emotion-oriented and avoidant problem-solving VS. Profile 2 Impulsivity-oriented and irrational problem-solving (ref)** | | | | |
| --- | --- | --- | --- | --- | --- |
|  | **OR** | **95% *CI*** | | | ***p*** |
| Gender (ref: female) |  |  | | |  |
| Male | 0.002 | 0.000–0.007 | | | <0.001*** |
| Age (ref: > 40) |  |  | | |  |
| < 30 | 1.603 | 0.143–17.974 | | | 0.702 |
| 30 - 40 | 1.109 | 0.278–4.422 | | | 0.884 |
| Marital status (ref: unmarried/widowed/divorced) |  |  | | |  |
| Married | 1.396 | 0.157–12.415 | | | 0.765 |
| Education level (ref: college) |  |  | | |  |
| Primary school | 2.795 | 0.414–18.887 | | | 0.292 |
| High school | 1.413 | 0.330–6.053 | | | 0.642 |
| Monthly income (¥, CNY^a^) (ref: >5000) |  |  |  |  | |
| < 3000 | 11.194 | 2.358–53.131 | | | 0.002** |
| 3000 - 5000 | 5.531 | 1.197–25.565 | | | 0.029* |
| Place of residence (ref: urban) |  |  |  |  | |
| Rural | 0.311 | 0.070–1.375 | | | 0.123 |
| Number of children (ref: ≥ 3) |  |  |  |  | |
| 1 | 0.698 | 0.106–4.582 | | | 0.708 |
| 2 | 2.054 | 0.413–10.215 | | | 0.379 |

Ref = Reference; SE = Standard error; OR = Odds ratio; ^a^CNY = China Yuan, US$ 1.00 = ¥ 7.02.

**p*  <  0.05 (2 - tailed); ***p* <  0.01 (2 - tailed); ****p* < 0.001 (2 - tailed).
